# Normal dimensions of the lacrimal gland on magnetic resonance imaging in Indian adult population: a retrospective study

**DOI:** 10.11604/pamj.2023.45.71.38213

**Published:** 2023-05-31

**Authors:** Vrushali Naresh Dalvi, Ashish Ambhore, Avinash Parshuram Dhok, Prashant Madhukarrao Onkar, Kajal Mitra

**Affiliations:** 1 Department of Radiodiagnosis and Imaging, NKP Salve Institute of Medical Sciences and Research Centre, Digdoh Hills, Nagpur 440019, Maharashtra, India

**Keywords:** Lacrimal gland, normal dimensions, magnetic resonance imaging, Indian population, retrospective study

## Abstract

**Introduction:**

the lacrimal gland size is affected by a variety of pathologic conditions like inflammatory, infections, neoplastic, autoimmune and granulomatous disorders. Earlier, the dimensions of the gland were estimated by extracting lacrimal glands from cadavers, later ultrasonography and computed tomography studies were used, but had limited soft tissue differentiation. The aim of this study was to retrospectively evaluate Magnetic Resonance Imaging (MRI) data from normal orbits and determine lacrimal gland dimensions.

**Methods:**

five hundred and twelve (512) consecutive MRI brain contrast scans (of 240 females, of 272 males; age range 40±20 years) for non-orbital diseases were evaluated retrospectively. The mean axial length (AL), axial width (AW), coronal length (CL), and coronal width (CW) of each lacrimal gland were measured separately.

**Results:**

five hundred and twelve (512) MRI brain contrast scans of 272 men and 240 women, with mean age of 40 ± 20 years were included. Right and left LG dimensions were similar, mean AL (13.2±1.35 mm versus 13.11±1.24 mm), mean AW [3.5±0.99 mm versus 3.3±0.82 mm], mean CL [16.3±2.5 mm versus 16.10±2.4 mm], and mean CW (4.15±0.89 mm versus 4.11±0.85 mm). The AL of both lacrimal glands and the CL and CW of right but not left lacrimal glands were significantly lower in women than in men. Age showed significant correlations with the AL and CL of both LGs.

**Conclusion:**

in this study, evaluation of normal morphometric parameters of the lacrimal gland in Indian population was established. LG dimensions are smaller in women than men, as well as correlating with side and age.

## Introduction

The lacrimal gland is an eccrine secretory gland that produces tears and is almond-shaped. It is situated on the orbit's superolateral side, abutting the superior and lateral rectus muscles. It is divided into two parts by the lateral horn of the levator palpebrae muscle's aponeurosis, which separates the orbital and palpebral lobes [1]. The lacrimal gland's orbital lobe is posterior and superior to the levator palpebrae aponeurosis, whereas the palpebral lobe is anterior and inferior. The orbital lobe is bigger and is where most epithelial neoplasms of the lacrimal gland occur. Infiltrative and inflammatory diseases frequently affect the gland's orbital and palpebral lobes [2]. On physical inspection, the palpebral lobe is usually apparent when the upper eyelid is everted. The lacrimal gland is usually about 20 x 12 x 5 mm in size. A normal lacrimal gland's size varies from person to person [2]. Asymmetry is a key sign of abnormalities in the lacrimal glands, which are generally symmetric [2]. The diagnosis of lacrimal gland diseases is typically difficult for doctors due to their vague appearance. A palpable mass or lump in the orbit's superolateral aspect is the most common symptom. The lacrimal gland is affected by a variety of variables ranging from physiological cause to inflammatory causes like Sjogren syndrome, Wegener granulomatosis, sarcoidosis to benign neoplastic lesions such as pleomorphic adenoma, ductal epithelial cysts, Warthin´s tumour as well as malignant tumours like carcinoma ex pleomorphic adenoma, adenoid cystic carcinoma and mucoepidermoid carcinoma [3]. The lacrimal gland can be affected by metastases, leukemic deposits, and lymphoma in rare cases. Primary lacrimal gland tumours are common, accounting for roughly 20% of all lacrimal gland tumours [4]. Aging, sex steroid hormone, pituitary-dependent factors, apoptosis, neurotrophic factors, immunological response, and infection are all thought to have a role in lacrimal gland pathology [5]. The lacrimal gland is affected by a variety of autoimmune and granulomatous disorders. As a result, changes in lacrimal gland size may aid in the diagnosis of these diseases [6]. Diseases of the lacrimal gland are often accompanied by variations in gland size, it is crucial to understand the typical dimensions and volume of the lacrimal gland. The size of the lacrimal glands might vary depending on age, gender, and ethnicity [7]. The examiner's experience is frequently used to ascertain the dimension of the lacrimal glands. Previously, the dimension of the gland was estimated by extracting lacrimal glands from cadavers, however, due to the tiny sample size and dissection's disruptive effect, this method was limited in practical use, which rendered the measurements less precise. Later ultrasonography and CT studies were used, however these studies were limited in their ability to differentiate soft tissue [8]. Nonetheless, the majority of these research studies are related largely to European samples with limited focus on the Indian population. If used for referencing, the use of erroneous values could lead to a delayed or unnecessary diagnosis. The soft tissue differentiation provided by MRI is excellent and hence images of the head and neck soft tissue, including the lacrimal gland [9]. As a result, the MRI modality can be utilised to diagnose and monitor lacrimal gland diseases [10]. This study aimed to determine the normal dimensions of lacrimal glands in the Indian adult population using MRI and also to determine the associations of lacrimal gland dimensions with age, sex and laterality. The objectives were to establish reference values for the normal size of lacrimal gland in Indian adult population.

## Methods

**Study design**: a retrospective study.

**Study setting**: department of radiodiagnosis of medical college and tertiary health care hospital in urban setting of central India.

**Study population**: patients who fulfilled the inclusion criteria were included in the study.

**Inclusion criteria**: MRI brain contrast scans done in Indian adult patients(with no specific ethnicity) and average age of 40±20 years without any prior history of orbital pathology or any present orbital complaints were included in the study.

**Exclusion criteria**: patients with history of previous or present orbital pathology or known autoimmune conditions were excluded. Incidental orbital pathologic findings found on present scans were excluded.

**Sample size**: it was calculated by including all consecutive MRI brain contrast scans done in previous 2 years and fulfilling the inclusion criteria which was 512 scans (of 272 males and of 240 females).

**Sampling technique**: a convenient method for sampling was used. All the consecutive MRI brain contrast scans of the patients fulfilling the inclusion criteria were evaluated retrospectively.

**Study protocol**: after the permission from the institutional ethical committee, a retrospective descriptive study was conducted by collecting data from MRI brain contrast scans performed with a 1.5 tesla scanner in age group of 40±20 years (without any orbital pathologies) in the previous two years´ duration was obtained and compiled.

**Figure 1:**
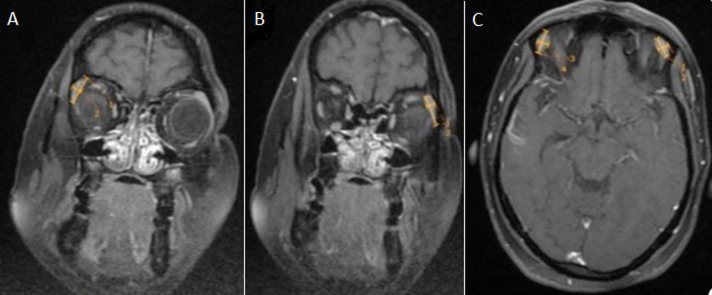
magnetic resonance imaging contrast-enhanced T1 weighted image of brain and orbit showing: A, B) coronal length and width of lacrimal gland on right and left sides; C) axial length and width of lacrimal gland on right and left sides

**Measurement of lacrimal gland**: in all the individuals, the contrast-enhanced fat-suppressed T1-weighted sequence MRI acquired in the axial and coronal planes with 1-mm contiguous sections over the whole brain were utilized (Figure 1) [1]. To determine the lacrimal gland's measurements, the axial and coronal planes were employed [1]. Axial length (AL) was determined from the extremes of the lacrimal gland's anterior and posterior aspect, and axial width (AW) was determined from the extremes of the gland's medial and lateral aspect on the axial plane. The extremes of medial and lateral edges of lacrimal gland served as the measuring point for coronal width (CW), whereas the extremes of superior and inferior tip served as the measurement point for coronal length (CL).

**Statistical analysis**: all information was gathered, recorded into an Excel spreadsheet, and considered confidential. The preferred tool for data analysis was IBM SPSS Statistics for Windows, version 24. (IBM Corp., Armonk, New York) The LG dimensions were compared using independent sample t-tests, and the Shapiro-Wilk test was used to assess whether the data were normally distributed. The link between age and the LG dimensions was studied using Pearson correlation tests [11]. Each subject's individual orbital side was analysed, and sex, age, and side analyses were performed on the parameters [11].

**Ethical approval**: the study was approved by Medical Ethics Committee of NKP Salve Institute of Medical Sciences and Research Centre with the letter number: [NKPSIMS & RC & LMH/IEC/07/2021].

## Results

This study included retrospective evaluation of MRI brain contrast scans of 512 participants, with 272 (53.1%) males and 240 [46.9%] women, with age ranging from 40±20 years and fulfilling the inclusion criteria. The mean AL, CL, AW, and CW measurements of the lacrimal glands on both sides were similar (Table 1). Contrarily, males and women's lacrimal gland size varied significantly (Table 2). Men's ALs were substantially longer than women's ALs in both the right (13.2±1.35 mm versus. 12.52±1.91 mm, p value=0.0010), and the left (13.11±1.24 mm versus, 12.12±1.7 mm, p value=0.001). Comparing males and women's right lacrimal glands revealed that men's mean CL (16.31±2.50 mm versus 15.34±1.98 mm, p value=0.001) and women's mean CW (4.15±0.89 mm versus 4.5±0.77 mm, p value=0.002) were substantially different from each other. On the other hand, mean coronal length and coronal width of the left lacrimal glands, as well as mean axial width on both sides, were comparable in both men and women. Our study showed that different lacrimal gland diameters significantly correlated with subject age (Table 3). Age strongly linked with the axial length (AL) (r coefficient= -0.321; p value <0.001), axial width (AW) (r coefficient= -0.454; p value <0.0010) of right lacrimal gland and AL (r coefficient= -0.344; p value <0.001), and AW (r coefficient=-0.451; p value <0.0010) of the left lacrimal glands. Contrarily, there was no discernible relationship between the subject's age and the coronal length and coronal width of either the right or left lacrimal gland.

**Table 1:** dimensions of right and left lacrimal glands in female and male subjects

Variables	Men (n=272)	Women (n=240)	p-value
**Right lacrimal gland**			
Axial length(AL) mm	13.2 ± 1.35	12.52 ± 1.91	0.001
Axial width (AW) mm	3.5 ±0.99	4.1±0.52	0.70
Coronal length(CL) mm	16.31 ± 2.5	15.34 ±1.98	0.001
Coronal width (CW) mm	4.15± 0.89	4.5±0.77	0.002
**Left lacrimal gland**			
Axial length(AL) mm	13.11 ± 1.24	12.12 ± 1.7	0.001
Axial width (AW) mm	3.3±0.82	3.8 ± 0.61	0.35
Coronal length(CL) mm	16.10 ± 2.4	15.01 ± 1.88	0.30
Coronal width (CW) mm	4.11± 0.85	4.1 ±0.75	0.95

**Table 2:** dimensions of the right and left lacrimal glands

Variables	Right lacrimal gland	Left lacrimal gland	P-value
Axial length(AL) mm	13.2 ± 1.35	13.11 ±1.24	0.43
Axial width (AW) mm	3.5 ± 0.99	3.3 ± 0.82	0.16
Coronal length(CL) mm	16.31 ± 2.5	16.10 ± 2.4	0.51
Coronal width (CW) mm	4.15 ± 0.89	4.11 ± 0.85	0.29

**Table 3:** correlation of age with dimensions of the lacrimal glands

Variables	Correlation coefficient (r)	P-value
**Right lacrimal gland**		
Axial length(AL) mm	-0.321	<0.001
Axial width (AW) mm	-0.454	<0.001
Coronal length(CL) mm	-0.042	0.69
Coronal width (CW) mm	0.047	0.71
**Left lacrimal gland**		
Axial length(AL) mm	-0.344	<0.001
Axial width (AW) mm	-0.451	<0.001
Coronal length(CL) mm	-0.051	0.70
Coronal width (CW) mm	0.052	0.68

## Discussion

The development of imaging technologies like computed tomography (CT) and magnetic resonance imaging (MRI) has significantly revolutionized salivary gland imaging. Because defining the morphology of the lacrimal gland on CT is significantly more challenging, MRI is used for lacrimal gland assessment. Because of the gland's hyperintense signal, the lacrimal gland outline is easier to distinguish on MRI post contrast images. An MRI, rather than exposing a patient to radiation, is indicated as the investigation of choice, particularly for their orbits; in worrisome circumstances, CT scans may be used as a diagnostic aid [1]. In the current study, the mean AL of the LG glands was 13.20 ±1.35 mm for the right lacrimal glands and 13.11±1.24 mm for the left lacrimal glands. The right and left lacrimal glands' average axial lengths in a Nigerian Study were 14.60 mm and 14.50 mm, respectively, comparatively the average axial length (AL) in the present study was less [10]. Mean coronal width for right and left lacrimal glands, respectively, were 4.15 mm and 4.10 mm in present study.

However, compared to a Nigerian study, who had mean CWs of 2.90 mm for the right LG and 3.00 mm for the left lacrimal gland, in the present study mean coronal width was greater. There were no significant variations in the dimensions of the LG between males and women subjects in the Nigerian research. LGs mean axial length were 13.2 ±1.35 and 12.52 ±1.91 mm on right side, respectively; mean coronal length for LGs were 16.31 ±2.5 and 15.34 ±1.98 mm respectively on right side; mean axial width was 3.5 ±0.99 and 4.1 ±0.52 mm, on right side and mean coronal width were 4.15 ±0.89 and 4.5 ±0.77 mm on right side. Similar measurements were observed for left lacrimal glands [10]. According to a research conducted on a Turkish study, the mean axial length, axial width, coronal length and coronal width measurements for right and left lacrimal glands, were 16.2, 4.1, 18.3, and 4.1 mm respectively [2]. In a Korean study, the mean axial length, axial width, coronal length and coronal width measurements for the right and left lacrimal glands, respectively, were 14.7, 5.1, 17.7, and 5.2 mm [3]. Table 4 provides a thorough comparison of lacrimal gland dimensions with those of other populations. Age was shown to substantially correlate with the axial length and axial width of both the right and left lacrimal glands, but not with the coronal length and coronal width of either lacrimal gland. Similar findings were observed in a Nigerian study, where age showed a minimal correlation with coronal width but a strong negative correlation with axial length, axial width and coronal length [10]. The limitation of our study is that a modest sample size was used and that it was done in a single facility so our results may not reflect dimensions of lacrimal glands in the wider Indian population. As a result, a large-scale, multicentre investigation is required to identify lacrimal gland dimensions. Only a few studies have been conducted to estimate the lacrimal gland size in Indians and the rest of the world. The biometry of the normal lacrimal gland can be used as a reference. Clinical and imaging data will be used to help diagnose various lacrimal gland diseases.

**Table 4:** comparison of right and left lacrimal gland dimensions in different other populations

	Present study	Turkish	Nigerians	Koreans	Caucasians
**Right lacrimal gland**					
Axial length(AL) mm	13.20	16.2	14.6	14.9	14.7
Axial width (AW) mm	3.50	4.1	4.1	4.1	5.1
Coronal length(CL) mm	16.31	18.3	20.7	20.9	16.9
Coronal width (CW) mm	4.15	4.1	2.9	3.6	5.2
**Left lacrimal gland**					
Axial length(AL) mm	13.11	16	14.5	14.7	14.5
Axial width (AW) mm	3.30	4.0	4.1	4.3	4.8
Coronal length(CL) mm	16.10	18.3	20.8	20.7	16.9
Coronal width (CW) mm	4.11	4.1	3.0	3.8	5.2

## Conclusion

The study's observations of lacrimal gland morphometric parameters are different from those found in earlier research that was conducted on western populations and appear to influence the threshold for deciding whether an individual has hypertrophy or atrophy of lacrimal gland in Indian population and hence indicates the necessity for substantial multicentre investigations to establish the same. The size and volume of the normal lacrimal gland can be used as a reference by the clinicians to assess gland size in various illnesses in the Indian adult population.

### What is known about this topic

- *Normal lacrimal gland dimensions studies have been conducted in western population;*
- *Computed tomography and ultrasonography modality were utilized for measurement of lacrimal gland morphometric parameters;*
- *Different lacrimal gland diameters significantly correlated with subject age*.

### What this study adds

- *Accurate lacrimal gland normal dimensions can be used as a reference in the Indian population;*
- *MRI contrast study which has excellent soft tissue differentiation is utilized for accurate measurement of the gland dimensions;*
- *Indian women have smaller size lacrimal glands as compared to men*.
